# Introducing women’s health in the higher education curriculum: An innovative experience in advancing women’s health

**DOI:** 10.15694/mep.2018.0000214.1

**Published:** 2018-09-18

**Authors:** Ghufran Jassim

**Affiliations:** 1 royal college of surgeons in ireland medical university of Bahrain

**Keywords:** Women’s health, curriculum, Bahrain, undergraduate, higher education, University for women

## Abstract

This article was migrated. The article was marked as recommended.

Women go through several stages and changes throughout their life time. Hence, their health needs differ according to their life stage. More importantly, women are perceived as the decision-makers for the source of health care for their families. The object of this project is to integrate women’s health into higher education curriculum with the aim of empowering women with the knowledge they need to take control of their own health through making informed health decisions, and to seek appropriate and timely care and managing the processes of illness and wellness. I designed an undergraduate course in women’s health that addresses the main health issues across the life span of women from local and global perspectives.I developed a stepwise process, or framework, for including women’s health course in the undergraduate curriculum of the Royal University for Women in the Kingdom of Bahrain. The course was offered as an elective module pertaining to 3 credits and comprising 3hours of teaching per week.

This pioneer experience of introducing women’s health to non-medical university students is innovative and supports the notion of “Healthy women hold up a healthy world”. Informed women ensure future informed health decisions. This experience will be evaluated and reflected upon with the hope of expanding it to other universities.

## Introduction

As defined by World Health Organization (WHO), health is a “State of complete physical, mental, and social wellbeing, and not merely the absence of disease or infirmity ( Nobile, 2014). Prevention is the foundation of the public health system. But we cannot begin to address prevention if our patients do not know what we are trying to say to them. Accordingly, improving the nation’s health literacy is critical to creating a system of care based on wellness and prevention ( Benjamin, 2010).

Women are perceived as the decision-makers for the source of health care for their families. In fact, women often delay self-care as they attend to the care of their family or children.

The relationship between health, gender equality and education has been extensively investigated. The existing literature sheds particular light on aspects related to literacy for young and adult women. On the one hand, it shows how the acquisition of literacy skills contributes to improving people’s health, while also fostering the empowerment of women. It is well documented that those who have had access to literacy and education tend to adopt healthier behaviours and have a greater measure of control over their bodies ( Robinson-Pant, 2016)

Women’s health has significantly evolved in the past two decades ( Karney, 2000). It has been merely seen as the health of the reproductive system. However, nowadays this shallow view is being replaced by a broader definition of women’s health. Women’s health is about recognising the diversity of women’s health issues at different stages of their lives. The fundamental principles are: encouraging women to take control of their bodies (based on a full range of information and access to appropriate health care), education (starting as early as possible), informed and shared decision making between women and their health care providers (with women deciding for themselves what happens to their bodies), and a social model of health (that recognises the context of women’s lives, e.g. the influence of social factors such as housing and employment on health and well-being) ( Norton, 2016).

According to the WHO the top ten health issues facing women are cancer, reproductive health, mental health, HIV, sexually transmitted infections, violence against women, maternal health, non-communicable diseases, being young and getting older ( Bustreo, 2015).

On the other hand, 60% of students in higher education in the Kingdom of Bahrain are female. This reflects a worldwide trend in higher education whereby over 50% of students are female ( David, 2015). Now, more than ever, health leaders recognize the critical need to integrate women’s health into universities curriculum.

## Methods

The process of incorporating new material into an existing curriculum frequently produces competition for curricular time. We developed a stepwise process, or framework, for including women’s health teaching for undergraduate students. The key elements of the process are to define the full scope of what needs to be taught, develop teaching objectives, identify opportunities to introduce the information into the curriculum, identify the key faculties, develop evaluation tools, assess whether students have achieved the expected competencies and evaluate the whole process.

### Curriculum development

We retrieved and reviewed undergraduate women’s health syllabi that were used in other institutions across the globe. Reviewing syllabi assisted in identifying the priorities in global and local women health issues to be included in the curriculum. We tailored the content to non-medical university students by using lay person, non-medical language. Course readings were retrieved from textbooks, peer-reviewed scientific c literature, news/ media articles and videos. They were incorporated into course readings to highlight local, national, and international women’s health perspectives.

Then we discussed where in the curriculum this can be embedded. There was a consensus that introducing the course as an elective is the best way of initiating this innovative concept.

### Setting

This innovative experience was implemented in the Royal University for Women (RUW). RUW is an accredited university established in 2005. It is the first private university in Bahrain dedicated solely to educating women. With 653 all-female students, the university has colleges of Art & Design, business, Information technology, Law and recently Civil Engineering.

The teaching objectives articulated the outcomes expected from students as a result of taking this course. They were grouped into 4 principle areas: Knowledge and understanding, subject specific skills, critical thinking skills, general and transferrable skills.

a. **Knowledge and Understanding**
  • Understand the concept and importance of women’s health.
  • Explain factors influencing women’s health
  • Identify the most common health issues facing women in local and global context
  • Identify different aspects of women’s health (metal, physical, emotional and psychological)
b. **Subject-Specific Skills**
  • Develop the skills in searching for the relevant evidence based information and apply it to women’s health issues.
  • Develop skills in identifying women’s health program that promote health and wellbeing.
c. **Critical Thinking Skills**
  • Express personal views regarding women’s health issues.
  • Critically analyze the evidence and information related to women’s health
  • Develop decision making skills in having control over one’s health
  • Develop skills in conducting scientific arguments about women’s health
d. **General and Transferable Skills**
  • Develop skills in oral and poster presentation
  • Use the best available resources in obtaining reliable information related to health

### Teaching faculties

The Royal University for Women identified a women health specialist (the author of this article) as the key faculty to deliver the content of the course. The identified instructor is a consultant family physician and women health specialist with over 10 years’ experience in higher education as a senior lecturer in the Royal College of Surgeons In Ireland- Medical University of Bahrain. The faculty developed the framework and the content of women’s health course as described in this article after a thorough search of the literature.

### Teaching methods

The course comprised of 16 lectures delivered using mixed teaching methods such as didactic lectures, case based teaching, flipped classroom, group discussion, student presentation, invited guest speakers and hands-on tasks.

The global context of women health was introduced through watching a short video on Maternal Mortality in Sierra Leone https://vimeo.com/48075598. After which students were requested to write a reflection pertaining to the video.

Guest speakers were invited to assist in extending content outside of the classroom. For example a nutritionist from Bahrain specialist hospital gave a detailed talk on practical nutritional tips and was well received by students. Another speaker is from the Supreme Counsel for women with the aim of describing the local legislations and efforts to enhance women’s health.

In an era of abundance online information, the hands-on tasks was introduced to evaluate the credibility of such online health information.

### Student assessment

The student evaluation consisted of 3 components: reflective assignment on the global women’s health video, mid-term evaluation (individual student presentation about any topic related to women’s health which did not necessarily have to be a medical condition) and a final written exam consisting of multiple choice questions and short essays (provide them with a stem/case study and request them to express their opinion in view of what they have learnt and read throughout the course).

## Results

### Course overview

The course was delivered in the two semesters of the academic year 2017/2018. It was offered as an elective module which carries 3 credits and comprising 3hours of teaching per week. In the first semester (where women health course is introduced for the very first time), 18 students have registered from different colleges with the majority being from years 2 and 3. In the second semester of the same academic year the number of enrolled students has risen to 38.

The content of the course is summarised in Table 1.

**Table 1.** List of lecture topics covered in the women’s health course

Introduction to women’s health
Global and local perspective of women’s health
Female growth and development and reproductive health
Most common women’s health issues Locally
Most common women’s health issues globally
Preventive programs in women’s health
Maternal and child health
Family planning
Nutrition and life style changes
Pregnancy and giving birth
Menopause
Eating disorder
Violence against women
Psychological health and common psychological conditions among women
Substance abuse
Sexually transmitted diseases

### Feedback on the course

Course evaluations were conducted formally (by the university administration) and informally (by the instructors). The formal student evaluation organised by the university showed a very positive response. The course was very well perceived and scored 18.96 out of 20 whereas the teacher evaluation achieved 19.13 out of 20 in the first semester.

The informal student evaluation conducted by the instructor focused on obtaining qualitative feedback from the course participants. Students appreciated very much the opportunity to learn about women’s health and to better understand the health issues that they could face across their life span. Students expressed their enthusiasm to attend lectures and transfer what they have learnt to their families and friends. Of particular interest, students valued the opportunity to learn about global health issues that they would never came across in the developed world. Also they liked the fact that the focus of the course was diverse and encompassed various aspects related to social and psychological wellbeing of women. Of greatest significance was students’ acknowledgement of how they themselves felt informed and equipped with skills and knowledge to make informed health decision regarding their health in conjunction with their health care providers.

## Discussion

Introducing women’s health in the curriculum of undergraduate university non-medical students is innovative. Women’s health is a multidimensional discipline with a breadth of topics. The course provided students with the knowledge and skills required to make informed decisions about their health in discussion with their health care providers. Such tools and skills are compatible with the notion of health literacy in which individuals have the capacity to obtain, process, and understand basic health information and services needed to make appropriate health decisions.

One of the limitations we faced while developing the curriculum is the scarce information available about syllabi from other similar courses in other institutions worldwide. This is mainly because most women’s health courses are introduced to students with some degree of medical knowledge for example: public health, dental and allied health professions students. This course was developed for undergraduate students who by enlarge have no background in medicine, nursing or any other health related studies. These students are studying Business, Marketing, Art, Law and engendering.

Although the language was “tuned down” by using non-medical, lay person terms to suit the audience, some students still felt that some topics and terms were a bit challenging to apprehend. This could be due to the fact that the course was delivered in English, when for most of not all students English was not the first language.

As a first trial of introducing a new concept in the existing curriculum, there is a lot of room for improvement. New topics can be added such as women with disabilities and women in the work force. Some topics might be better separated to allow more time for discussion such as the nutrition and life style lecture to be split and expanded further into body image and physical fitness lectures. Also there is a considerable value in introducing debates group of controversial women’s health issues for example abortion and expanding it to incorporate legal and social aspects since there are students from the law school.

Another future planning is to partner with other schools in a transdisciplinary approach to identify ways of linking women’s health topics with other concepts in the curriculum for example legislations on women’s health issues (Law school), economic perspectives of women’s health (business and marketing) and inspiring Art students in their projects. This transdisciplinary approach will provide additional richness and ideal framework to view women’s health as a holistic concept encompassing various aspects of women’s life.

## Take Home Messages

• Educating women about their health should start as early as possible.
• The high health returns to investing in the education of women are indisputable.
• Well educated women can make informed decisions regarding their health and the health of their loved ones.
• Improving the nation’s health literacy is critical to creating a system of care based on wellness and prevention.
• This pioneer experience of introducing women’s health to non-medical university students is innovative and supports the notion of “Healthy women hold up a healthy world”.

## Notes On Contributors

The author is a senior lecturer at the Royal College of Surgeons in Ireland Medical University of Bahrain. She graduated in Medicine and completed a family residency program. She was awarded a PhD degree in General Practice from the Royal College of Surgeons in Ireland. She has a diploma in medical education profession and completed a year progran in Global Clinical Scholars Research Training at Harvard Medical School - Harvard University. Her research interests are child and women health, breast cancer, non communicable diseases and evidence based medicine. She has published many articles in International peer reviewed journals and presented in many international and local conferences.
