# Supplementary material for: Introducing women’s health in the higher education curriculum: An innovative experience in advancing women’s health
Source: MedEdPublish (2016). 2018 Sep 18;7:214. [Version 1] doi: 10.15694/mep.2018.0000214.1 (PMC10704443; doi:10.15694/mep.2018.0000214.1)
Supplement: Peer review history

# Reviewer report: Reviewer response for version 1

**DOI:** 10.21956/mep.19671.r29376

**Published:** 2018-10-10

**Authors:** Ariela Marshall

**Affiliations:** 1 Mayo Clinic

This review has been migrated. The reviewer awarded 4 stars out of 5 I commend the author on the successful implementation of a women’s health curriculum at her medical center. This is certainly an area of need and the author has designed a comprehensive program to address deficiencies in the existing curriculum and address issues which are crucially important to the health and well-being not only of women but the community as a whole.I have a few recommendations for the author that may further strengthen the paper:1. Some of the language and sentence structure is difficult to follow. For example, the third paragraph of the introduction includes the phrase “on the one hand” but the follow-up phrase “on the other hand” does not appear until the 6th paragraph of the introduction and the counter-argument does not appear to flow easily from the first “on the one hand.” This is just one example of several areas where the grammar/sentence structure could be improved2. It would be helpful to include details of the curriculum for each of the topics listed in Table 1; even 1-2 bullet points of the major teaching points/learning objectives for each topic would be educational for the readers3. The “assessment” section of methods would benefit from further details. The student evaluation included reflective assessment – was this written? The mid-term included a presentation – was the presentation oral, written or both? The final examination consisted of multiple choice questions and short answer essays – how many of each and what was the scoring system (1-10, etc)?4. The “feedback on the course” section of results would also benefit from further details. How were formal evaluations structured? What was the score of 20 based on? Was it a visual scale, etc, and what were the anchors on either end of the scale?

Reviewer Expertise:

NA

No decision status is available

# Reviewer report: Reviewer response for version 1

**DOI:** 10.21956/mep.19671.r29375

**Published:** 2018-09-21

**Authors:** Michelle McLean

**Affiliations:** 1 Bond University

This review has been migrated. The reviewer awarded 4 stars out of 5 I found this article easy to read as it was a descriptive summary of an elective course introduced into a female-only university in the Middle East. I work with medical students and one would think that they would be curious to know how their bodies work. Imaging the anatomy and physiology to their own bodies should make learning easy but this is not the case. Most can't really apply what they are learning to their own structure and function. Thus, offering a course to non-medical women, who would know even less than medical students, is a great idea. As the author points out, women are often the 'face' of the family and so being able to understand some of the common health issues facing women locally and globally would be liberating. Such informed women then become a support network for others. Equally important, however, is the neglected issue of men's health. The research tells us that men are less likely to visit a doctor if they are feeling unwell, brushing it aside until it is too late. Developing such a course for male students at universities might go a long way to making them aware of issues facing men. The framework can be easily replicated and modified for local needs.

Reviewer Expertise:

NA

No decision status is available
